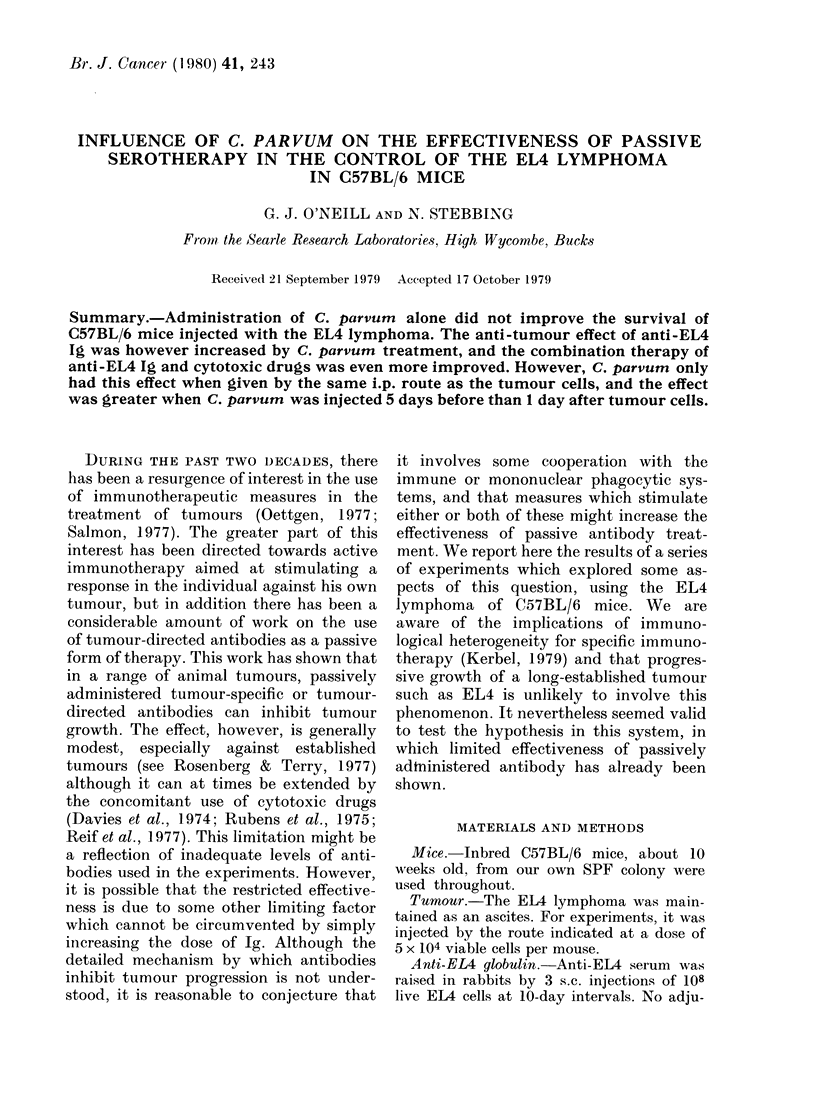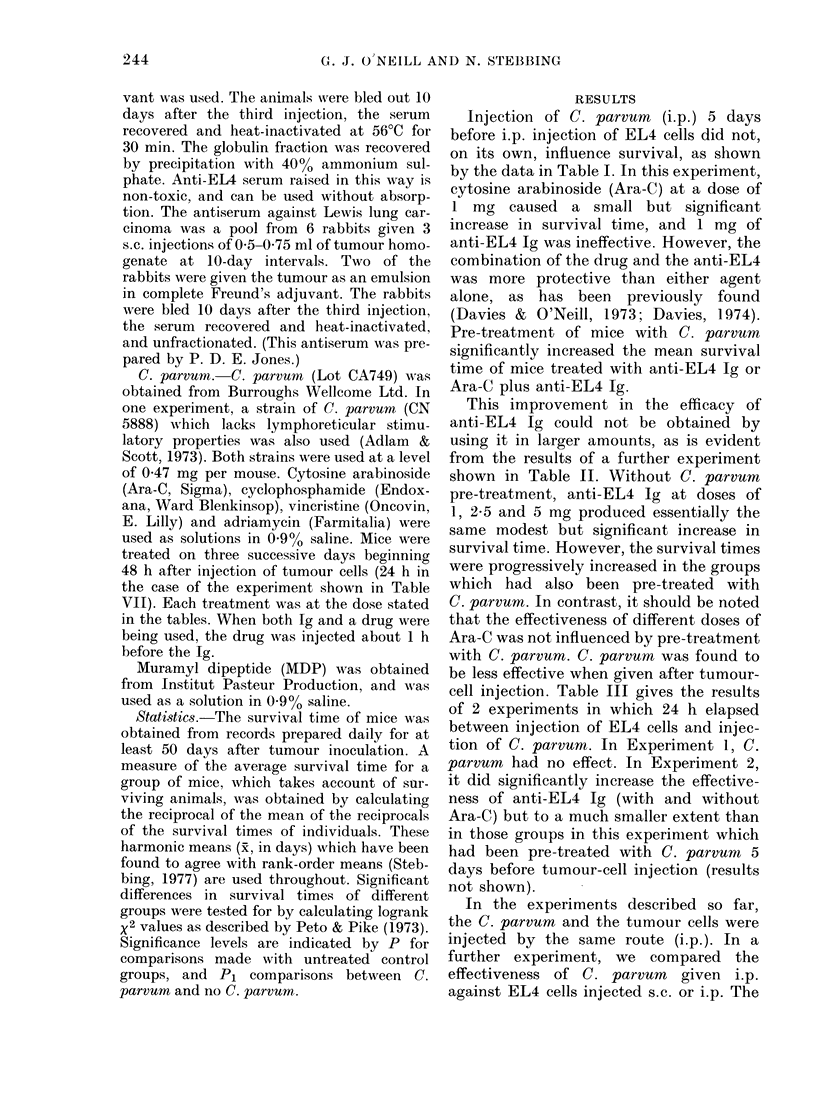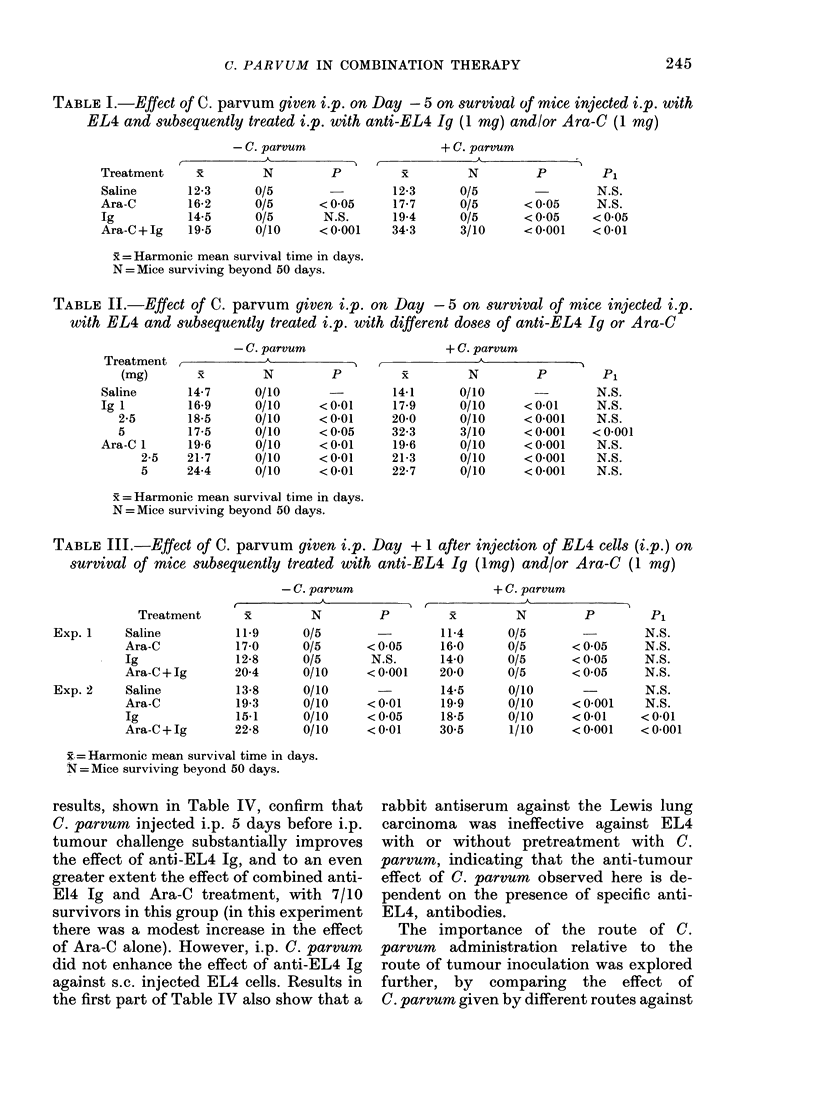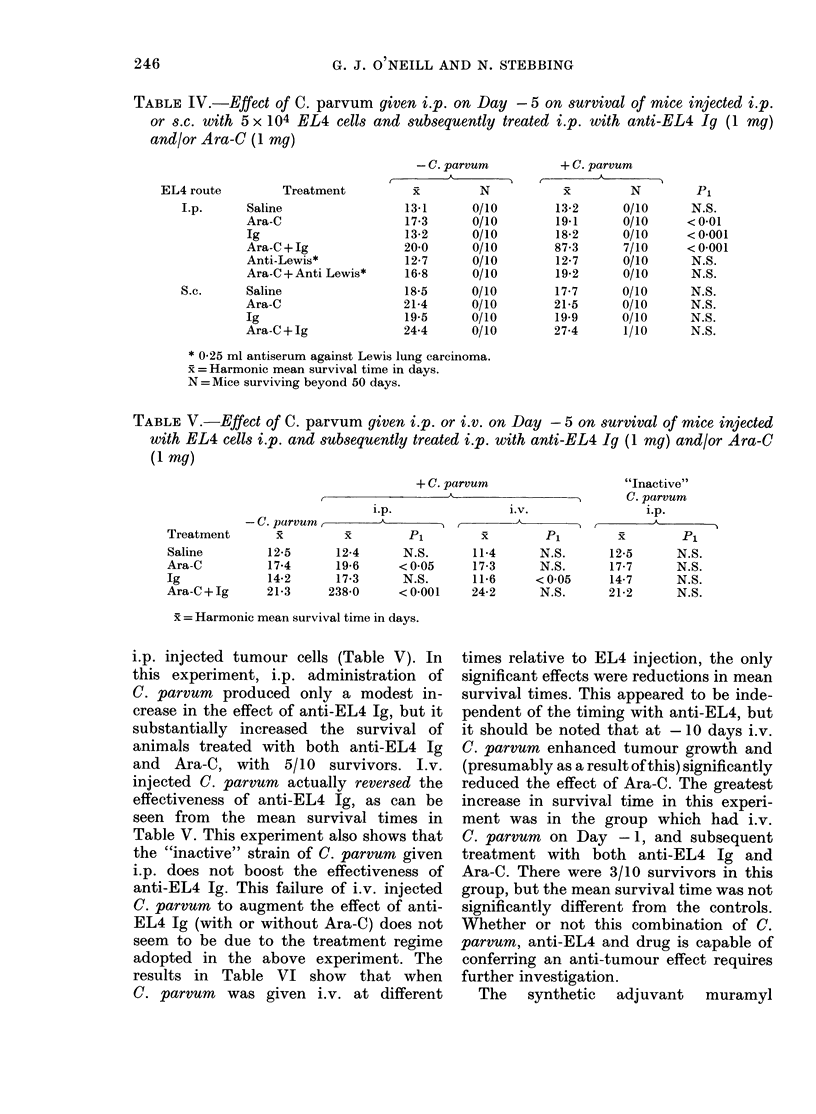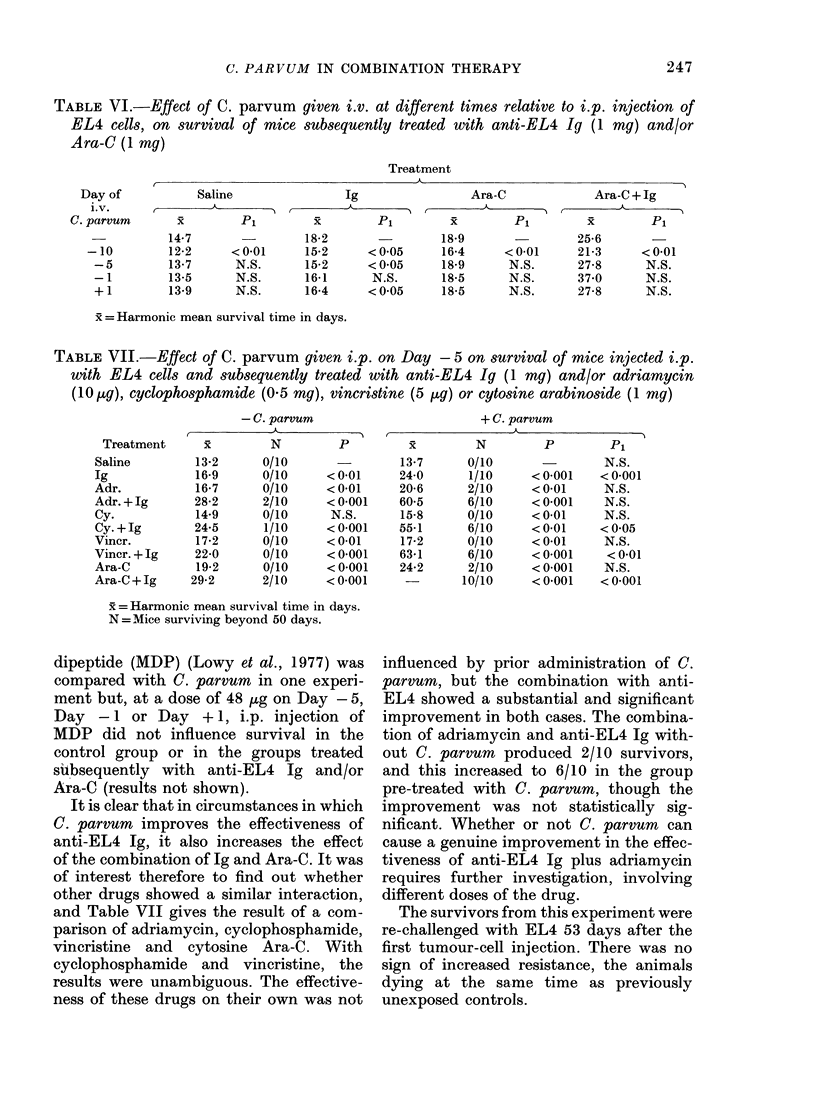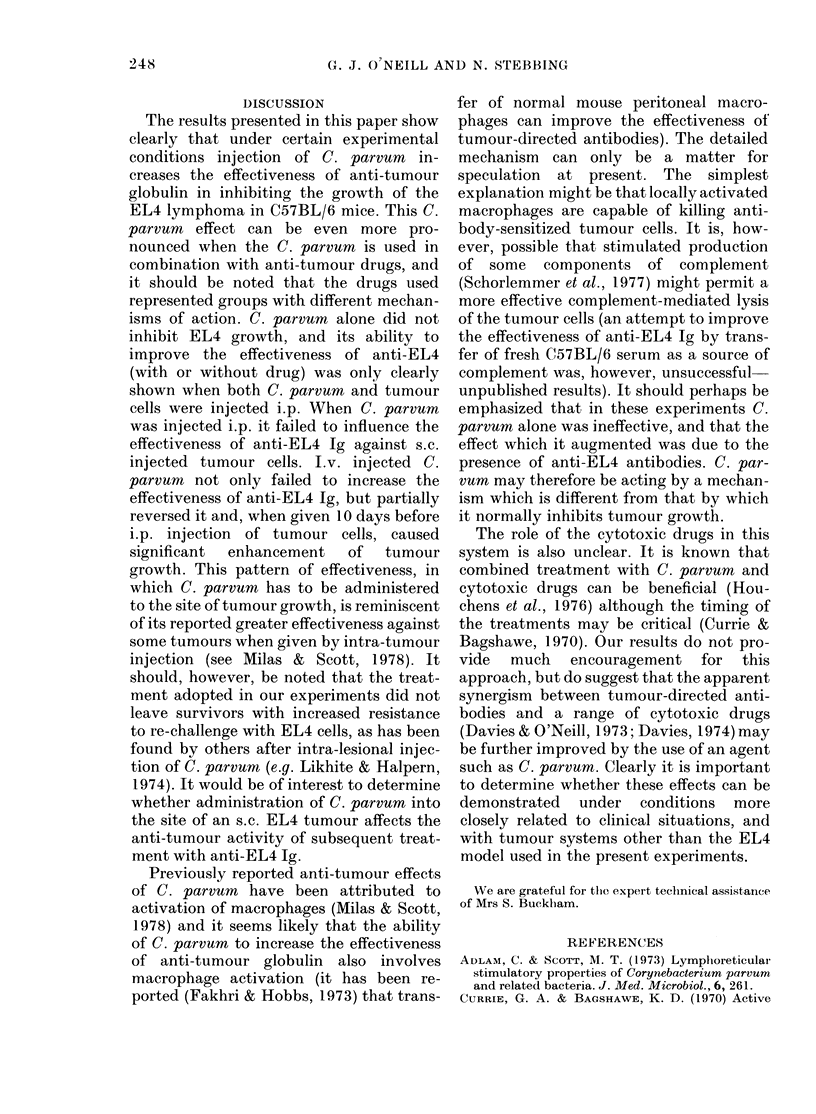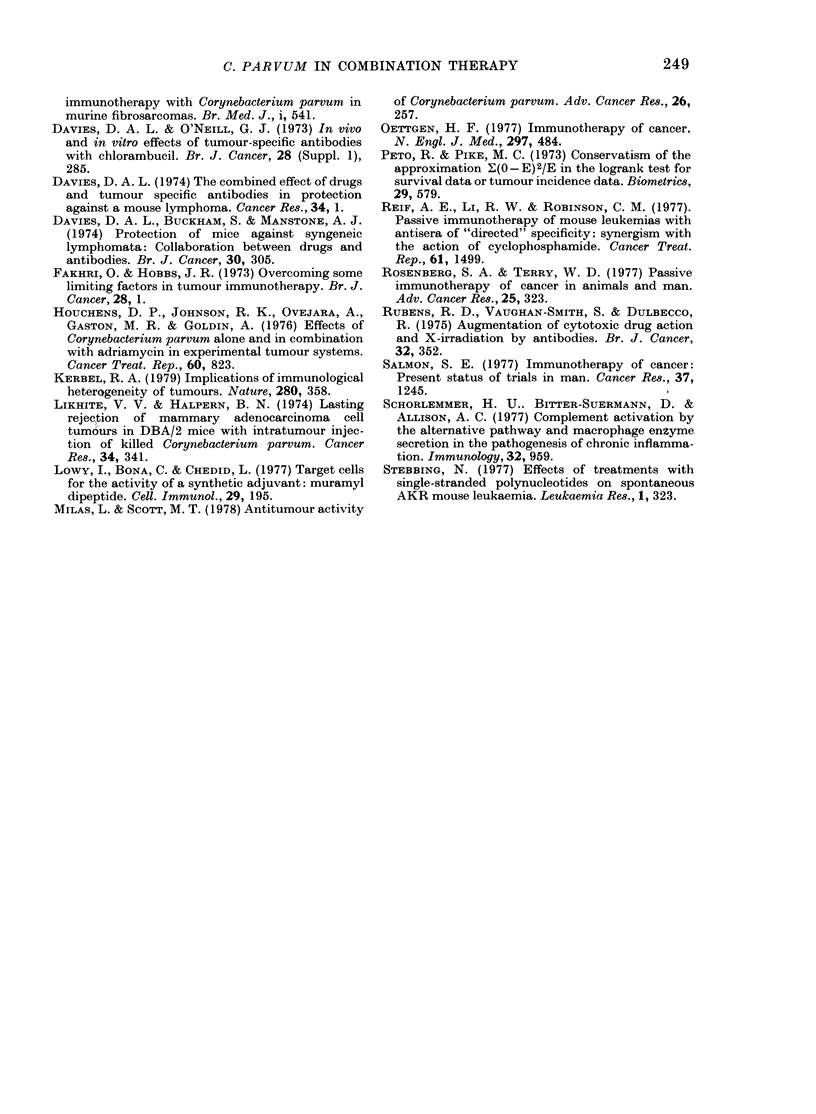# Influence of C. parvum on the effectiveness of passive serotherapy in the control of the EL4 lymphoma in C57BL/6 mice.

**DOI:** 10.1038/bjc.1980.36

**Published:** 1980-02

**Authors:** G. J. O'Neill, N. Stebbing

## Abstract

Administration of C. parvum alone did not improve the survival of C57BL/6 mice injected with the EL4 lymphoma. The anti-tumour effect of anti-EL4 Ig was however increased by C. parvum treatment, and the combination therapy of anti-EL4 Ig and cytotoxic drugs was even more improved. However, C. parvum only had this effect when given by the same i.p. route as the tumour cells, and the effect was greater when C. parvum was injected 5 days before than 1 day after tumour cells.


*Br. J. Cancer* (1980) **41**, 243

### INFLUENCE OF *C. PARVUM* ON THE EFFECTIVENESS OF PASSIVE SEROTHERAPY IN THE CONTROL OF THE EL4 LYMPHOMA IN C57BL/6 MICE

G. J. O'NEILL AND N. STEBBING

*From the Searle Research Laboratories, High Wycombe, Bucks*

Received 21 September 1979 Accepted 17 October 1979

**Summary.—Administration of *C. parvum* alone did not improve the survival of C57BL/6 mice injected with the EL4 lymphoma. The anti-tumour effect of anti-EL4 Ig was however increased by *C. parvum* treatment, and the combination therapy of anti-EL4 Ig and cytotoxic drugs was even more improved. However, *C. parvum* only had this effect when given by the same i.p. route as the tumour cells, and the effect was greater when *C. parvum* was injected 5 days before than 1 day after tumour cells.**

DURING THE PAST TWO DECADES, there has been a resurgence of interest in the use of immunotherapeutic measures in the treatment of tumours (Oettgen, 1977; Salmon, 1977). The greater part of this interest has been directed towards active immunotherapy aimed at stimulating a response in the individual against his own tumour, but in addition there has been a considerable amount of work on the use of tumour-directed antibodies as a passive form of therapy. This work has shown that in a range of animal tumours, passively administered tumour-specific or tumour-directed antibodies can inhibit tumour growth. The effect, however, is generally modest, especially against established tumours (see Rosenberg & Terry, 1977) although it can at times be extended by the concomitant use of cytotoxic drugs (Davies *et al.*, 1974; Rubens *et al.*, 1975; Reif *et al.*, 1977). This limitation might be a reflection of inadequate levels of antibodies used in the experiments. However, it is possible that the restricted effectiveness is due to some other limiting factor which cannot be circumvented by simply increasing the dose of Ig. Although the detailed mechanism by which antibodies inhibit tumour progression is not understood, it is reasonable to conjecture that it involves some cooperation with the immune or mononuclear phagocytic systems, and that measures which stimulate either or both of these might increase the effectiveness of passive antibody treatment. We report here the results of a series of experiments which explored some aspects of this question, using the EL4 lymphoma of C57BL/6 mice. We are aware of the implications of immunological heterogeneity for specific immunotherapy (Kerbel, 1979) and that progressive growth of a long-established tumour such as EL4 is unlikely to involve this phenomenon. It nevertheless seemed valid to test the hypothesis in this system, in which limited effectiveness of passively administered antibody has already been shown.

### MATERIALS AND METHODS

*Mice.*—Inbred C57BL/6 mice, about 10 weeks old, from our own SPF colony were used throughout.

*Tumour.*—The EL4 lymphoma was maintained as an ascites. For experiments, it was injected by the route indicated at a dose of $5 \times 10^4$ viable cells per mouse.

*Anti-EL4 globulin.*—Anti-EL4 serum was raised in rabbits by 3 s.c. injections of $10^8$ live EL4 cells at 10-day intervals. No adjuvant was used. The animals were bled out 10 days after the third injection, the serum recovered and heat-inactivated at 56°C for 30 min. The globulin fraction was recovered by precipitation with 40% ammonium sulphate. Anti-EL4 serum raised in this way is non-toxic, and can be used without absorption. The antiserum against Lewis lung carcinoma was a pool from 6 rabbits given 3 s.c. injections of 0·5–0·75 ml of tumour homogenate at 10-day intervals. Two of the rabbits were given the tumour as an emulsion in complete Freund's adjuvant. The rabbits were bled 10 days after the third injection, the serum recovered and heat-inactivated, and unfractionated. (This antiserum was prepared by P. D. E. Jones.)

*C. parvum*.—*C. parvum* (Lot CA749) was obtained from Burroughs Wellcome Ltd. In one experiment, a strain of *C. parvum* (CN 5888) which lacks lymphoreticular stimulatory properties was also used (Adlam & Scott, 1973). Both strains were used at a level of 0·47 mg per mouse. Cytosine arabinoside (Ara-C, Sigma), cyclophosphamide (Endoxana, Ward Blenkinsop), vincristine (Oncovin, E. Lilly) and adriamycin (Farmitalia) were used as solutions in 0·9% saline. Mice were treated on three successive days beginning 48 h after injection of tumour cells (24 h in the case of the experiment shown in Table VII). Each treatment was at the dose stated in the tables. When both Ig and a drug were being used, the drug was injected about 1 h before the Ig.

Muramyl dipeptide (MDP) was obtained from Institut Pasteur Production, and was used as a solution in 0·9% saline.

*Statistics*.—The survival time of mice was obtained from records prepared daily for at least 50 days after tumour inoculation. A measure of the average survival time for a group of mice, which takes account of surviving animals, was obtained by calculating the reciprocal of the mean of the reciprocals of the survival times of individuals. These harmonic means ($\bar{x}$, in days) which have been found to agree with rank-order means (Stebbing, 1977) are used throughout. Significant differences in survival times of different groups were tested for by calculating logrank $\chi^2$ values as described by Peto & Pike (1973). Significance levels are indicated by $P$ for comparisons made with untreated control groups, and $P_1$ comparisons between *C. parvum* and no *C. parvum*.

### RESULTS

Injection of *C. parvum* (i.p.) 5 days before i.p. injection of EL4 cells did not, on its own, influence survival, as shown by the data in Table I. In this experiment, cytosine arabinoside (Ara-C) at a dose of 1 mg caused a small but significant increase in survival time, and 1 mg of anti-EL4 Ig was ineffective. However, the combination of the drug and the anti-EL4 was more protective than either agent alone, as has been previously found (Davies & O'Neill, 1973; Davies, 1974). Pre-treatment of mice with *C. parvum* significantly increased the mean survival time of mice treated with anti-EL4 Ig or Ara-C plus anti-EL4 Ig.

This improvement in the efficacy of anti-EL4 Ig could not be obtained by using it in larger amounts, as is evident from the results of a further experiment shown in Table II. Without *C. parvum* pre-treatment, anti-EL4 Ig at doses of 1, 2·5 and 5 mg produced essentially the same modest but significant increase in survival time. However, the survival times were progressively increased in the groups which had also been pre-treated with *C. parvum*. In contrast, it should be noted that the effectiveness of different doses of Ara-C was not influenced by pre-treatment with *C. parvum*. *C. parvum* was found to be less effective when given after tumour-cell injection. Table III gives the results of 2 experiments in which 24 h elapsed between injection of EL4 cells and injection of *C. parvum*. In Experiment 1, *C. parvum* had no effect. In Experiment 2, it did significantly increase the effectiveness of anti-EL4 Ig (with and without Ara-C) but to a much smaller extent than in those groups in this experiment which had been pre-treated with *C. parvum* 5 days before tumour-cell injection (results not shown).

In the experiments described so far, the *C. parvum* and the tumour cells were injected by the same route (i.p.). In a further experiment, we compared the effectiveness of *C. parvum* given i.p. against EL4 cells injected s.c. or i.p. The

TABLE I.—*Effect of* C. parvum *given i.p. on Day* −5 *on survival of mice injected i.p. with EL4 and subsequently treated i.p. with anti-EL4 Ig* (1 *mg*) *and/or Ara-C* (1 *mg*)

| Treatment | − C. parvum x̄ | N | *P* | + C. parvum x̄ | N | *P* | $P_1$ |
|---|---|---|---|---|---|---|---|
| Saline | 12·3 | 0/5 | — | 12·3 | 0/5 | — | N.S. |
| Ara-C | 16·2 | 0/5 | < 0·05 | 17·7 | 0/5 | < 0·05 | N.S. |
| Ig | 14·5 | 0/5 | N.S. | 19·4 | 0/5 | < 0·05 | < 0·05 |
| Ara-C + Ig | 19·5 | 0/10 | < 0·001 | 34·3 | 3/10 | < 0·001 | < 0·01 |

x̄ = Harmonic mean survival time in days.
N = Mice surviving beyond 50 days.

TABLE II.—*Effect of* C. parvum *given i.p. on Day* −5 *on survival of mice injected i.p. with EL4 and subsequently treated i.p. with different doses of anti-EL4 Ig or Ara-C*

| Treatment (mg) | − C. parvum x̄ | N | *P* | + C. parvum x̄ | N | *P* | $P_1$ |
|---|---|---|---|---|---|---|---|
| Saline | 14·7 | 0/10 | — | 14·1 | 0/10 | — | N.S. |
| Ig 1 | 16·9 | 0/10 | < 0·01 | 17·9 | 0/10 | < 0·01 | N.S. |
| 2·5 | 18·5 | 0/10 | < 0·01 | 20·0 | 0/10 | < 0·001 | N.S. |
| 5 | 17·5 | 0/10 | < 0·05 | 32·3 | 3/10 | < 0·001 | < 0·001 |
| Ara-C 1 | 19·6 | 0/10 | < 0·01 | 19·6 | 0/10 | < 0·001 | N.S. |
| 2·5 | 21·7 | 0/10 | < 0·01 | 21·3 | 0/10 | < 0·001 | N.S. |
| 5 | 24·4 | 0/10 | < 0·01 | 22·7 | 0/10 | < 0·001 | N.S. |

x̄ = Harmonic mean survival time in days.
N = Mice surviving beyond 50 days.

TABLE III.—*Effect of* C. parvum *given i.p. Day* + 1 *after injection of EL4 cells* (*i.p.*) *on survival of mice subsequently treated with anti-EL4 Ig* (1*mg*) *and/or Ara-C* (1 *mg*)

| | Treatment | − C. parvum x̄ | N | *P* | + C. parvum x̄ | N | *P* | $P_1$ |
|---|---|---|---|---|---|---|---|---|
| Exp. 1 | Saline | 11·9 | 0/5 | — | 11·4 | 0/5 | — | N.S. |
| | Ara-C | 17·0 | 0/5 | < 0·05 | 16·0 | 0/5 | < 0·05 | N.S. |
| | Ig | 12·8 | 0/5 | N.S. | 14·0 | 0/5 | < 0·05 | N.S. |
| | Ara-C + Ig | 20·4 | 0/10 | < 0·001 | 20·0 | 0/5 | < 0·05 | N.S. |
| Exp. 2 | Saline | 13·8 | 0/10 | — | 14·5 | 0/10 | — | N.S. |
| | Ara-C | 19·3 | 0/10 | < 0·01 | 19·9 | 0/10 | < 0·001 | N.S. |
| | Ig | 15·1 | 0/10 | < 0·05 | 18·5 | 0/10 | < 0·01 | < 0·01 |
| | Ara-C + Ig | 22·8 | 0/10 | < 0·01 | 30·5 | 1/10 | < 0·001 | < 0·001 |

x̄ = Harmonic mean survival time in days.
N = Mice surviving beyond 50 days.

results, shown in Table IV, confirm that *C. parvum* injected i.p. 5 days before i.p. tumour challenge substantially improves the effect of anti-EL4 Ig, and to an even greater extent the effect of combined anti-El4 Ig and Ara-C treatment, with 7/10 survivors in this group (in this experiment there was a modest increase in the effect of Ara-C alone). However, i.p. *C. parvum* did not enhance the effect of anti-EL4 Ig against s.c. injected EL4 cells. Results in the first part of Table IV also show that a rabbit antiserum against the Lewis lung carcinoma was ineffective against EL4 with or without pretreatment with *C. parvum*, indicating that the anti-tumour effect of *C. parvum* observed here is dependent on the presence of specific anti-EL4, antibodies.

The importance of the route of *C. parvum* administration relative to the route of tumour inoculation was explored further, by comparing the effect of *C. parvum* given by different routes against

TABLE IV.—*Effect of* C. parvum *given i.p. on Day* −5 *on survival of mice injected i.p. or s.c. with* $5\times10^4$ *EL4 cells and subsequently treated i.p. with anti-EL4 Ig* (1 *mg*) *and/or Ara-C* (1 *mg*)

| EL4 route | Treatment | −*C. parvum* x̄ | −*C. parvum* N | +*C. parvum* x̄ | +*C. parvum* N | $P_1$ |
|---|---|---|---|---|---|---|
| I.p. | Saline | 13·1 | 0/10 | 13·2 | 0/10 | N.S. |
| | Ara-C | 17·3 | 0/10 | 19·1 | 0/10 | <0·01 |
| | Ig | 13·2 | 0/10 | 18·2 | 0/10 | <0·001 |
| | Ara-C+Ig | 20·0 | 0/10 | 87·3 | 7/10 | <0·001 |
| | Anti-Lewis* | 12·7 | 0/10 | 12·7 | 0/10 | N.S. |
| | Ara-C+Anti Lewis* | 16·8 | 0/10 | 19·2 | 0/10 | N.S. |
| S.c. | Saline | 18·5 | 0/10 | 17·7 | 0/10 | N.S. |
| | Ara-C | 21·4 | 0/10 | 21·5 | 0/10 | N.S. |
| | Ig | 19·5 | 0/10 | 19·9 | 0/10 | N.S. |
| | Ara-C+Ig | 24·4 | 0/10 | 27·4 | 1/10 | N.S. |

* 0·25 ml antiserum against Lewis lung carcinoma.
x̄ = Harmonic mean survival time in days.
N = Mice surviving beyond 50 days.

TABLE V.—*Effect of* C. parvum *given i.p. or i.v. on Day* −5 *on survival of mice injected with EL4 cells i.p. and subsequently treated i.p. with anti-EL4 Ig* (1 *mg*) *and/or Ara-C* (1 *mg*)

| Treatment | −*C. parvum* x̄ | +*C. parvum* i.p. x̄ | +*C. parvum* i.p. $P_1$ | +*C. parvum* i.v. x̄ | +*C. parvum* i.v. $P_1$ | "Inactive" *C. parvum* i.p. x̄ | "Inactive" *C. parvum* i.p. $P_1$ |
|---|---|---|---|---|---|---|---|
| Saline | 12·5 | 12·4 | N.S. | 11·4 | N.S. | 12·5 | N.S. |
| Ara-C | 17·4 | 19·6 | <0·05 | 17·3 | N.S. | 17·7 | N.S. |
| Ig | 14·2 | 17·3 | N.S. | 11·6 | <0·05 | 14·7 | N.S. |
| Ara-C+Ig | 21·3 | 238·0 | <0·001 | 24·2 | N.S. | 21·2 | N.S. |

x̄ = Harmonic mean survival time in days.

i.p. injected tumour cells (Table V). In this experiment, i.p. administration of *C. parvum* produced only a modest increase in the effect of anti-EL4 Ig, but it substantially increased the survival of animals treated with both anti-EL4 Ig and Ara-C, with 5/10 survivors. I.v. injected *C. parvum* actually *reversed* the effectiveness of anti-EL4 Ig, as can be seen from the mean survival times in Table V. This experiment also shows that the "inactive" strain of *C. parvum* given i.p. does not boost the effectiveness of anti-EL4 Ig. This failure of i.v. injected *C. parvum* to augment the effect of anti-EL4 Ig (with or without Ara-C) does not seem to be due to the treatment regime adopted in the above experiment. The results in Table VI show that when *C. parvum* was given i.v. at different times relative to EL4 injection, the only significant effects were reductions in mean survival times. This appeared to be independent of the timing with anti-EL4, but it should be noted that at −10 days i.v. *C. parvum* enhanced tumour growth and (presumably as a result of this) significantly reduced the effect of Ara-C. The greatest increase in survival time in this experiment was in the group which had i.v. *C. parvum* on Day −1, and subsequent treatment with both anti-EL4 Ig and Ara-C. There were 3/10 survivors in this group, but the mean survival time was not significantly different from the controls. Whether or not this combination of *C. parvum*, anti-EL4 and drug is capable of conferring an anti-tumour effect requires further investigation.

The synthetic adjuvant muramyl

TABLE VI.—*Effect of* C. parvum *given i.v. at different times relative to i.p. injection of EL4 cells, on survival of mice subsequently treated with anti-EL4 Ig* (1 *mg*) *and/or Ara-C* (1 *mg*)

| Day of i.v. *C. parvum* | Treatment: Saline $\bar{x}$ | Saline $P_1$ | Ig $\bar{x}$ | Ig $P_1$ | Ara-C $\bar{x}$ | Ara-C $P_1$ | Ara-C + Ig $\bar{x}$ | Ara-C + Ig $P_1$ |
|---|---|---|---|---|---|---|---|---|
| — | 14·7 | — | 18·2 | — | 18·9 | — | 25·6 | — |
| −10 | 12·2 | < 0·01 | 15·2 | < 0·05 | 16·4 | < 0·01 | 21·3 | < 0·01 |
| −5 | 13·7 | N.S. | 15·2 | < 0·05 | 18·9 | N.S. | 27·8 | N.S. |
| −1 | 13·5 | N.S. | 16·1 | N.S. | 18·5 | N.S. | 37·0 | N.S. |
| +1 | 13·9 | N.S. | 16·4 | < 0·05 | 18·5 | N.S. | 27·8 | N.S. |

$\bar{x}$ = Harmonic mean survival time in days.

TABLE VII.—*Effect of* C. parvum *given i.p. on Day −5 on survival of mice injected i.p. with EL4 cells and subsequently treated with anti-EL4 Ig* (1 *mg*) *and/or adriamycin* (10 *μg*), *cyclophosphamide* (0·5 *mg*), *vincristine* (5 *μg*) *or cytosine arabinoside* (1 *mg*)

| Treatment | − *C. parvum* $\bar{x}$ | N | *P* | + *C. parvum* $\bar{x}$ | N | *P* | $P_1$ |
|---|---|---|---|---|---|---|---|
| Saline | 13·2 | 0/10 | — | 13·7 | 0/10 | — | N.S. |
| Ig | 16·9 | 0/10 | < 0·01 | 24·0 | 1/10 | < 0·001 | < 0·001 |
| Adr. | 16·7 | 0/10 | < 0·01 | 20·6 | 2/10 | < 0·01 | N.S. |
| Adr. + Ig | 28·2 | 2/10 | < 0·001 | 60·5 | 6/10 | < 0·001 | N.S. |
| Cy. | 14·9 | 0/10 | N.S. | 15·8 | 0/10 | < 0·01 | N.S. |
| Cy. + Ig | 24·5 | 1/10 | < 0·001 | 55·1 | 6/10 | < 0·01 | < 0·05 |
| Vincr. | 17·2 | 0/10 | < 0·01 | 17·2 | 0/10 | < 0·01 | N.S. |
| Vincr. + Ig | 22·0 | 0/10 | < 0·001 | 63·1 | 6/10 | < 0·001 | < 0·01 |
| Ara-C | 19·2 | 0/10 | < 0·001 | 24·2 | 2/10 | < 0·001 | N.S. |
| Ara-C + Ig | 29·2 | 2/10 | < 0·001 | — | 10/10 | < 0·001 | < 0·001 |

$\bar{x}$ = Harmonic mean survival time in days.
N = Mice surviving beyond 50 days.

dipeptide (MDP) (Lowy *et al.*, 1977) was compared with *C. parvum* in one experiment but, at a dose of 48 μg on Day −5, Day −1 or Day +1, i.p. injection of MDP did not influence survival in the control group or in the groups treated subsequently with anti-EL4 Ig and/or Ara-C (results not shown).

It is clear that in circumstances in which *C. parvum* improves the effectiveness of anti-EL4 Ig, it also increases the effect of the combination of Ig and Ara-C. It was of interest therefore to find out whether other drugs showed a similar interaction, and Table VII gives the result of a comparison of adriamycin, cyclophosphamide, vincristine and cytosine Ara-C. With cyclophosphamide and vincristine, the results were unambiguous. The effectiveness of these drugs on their own was not influenced by prior administration of *C. parvum*, but the combination with anti-EL4 showed a substantial and significant improvement in both cases. The combination of adriamycin and anti-EL4 Ig without *C. parvum* produced 2/10 survivors, and this increased to 6/10 in the group pre-treated with *C. parvum*, though the improvement was not statistically significant. Whether or not *C. parvum* can cause a genuine improvement in the effectiveness of anti-EL4 Ig plus adriamycin requires further investigation, involving different doses of the drug.

The survivors from this experiment were re-challenged with EL4 53 days after the first tumour-cell injection. There was no sign of increased resistance, the animals dying at the same time as previously unexposed controls.

### DISCUSSION

The results presented in this paper show clearly that under certain experimental conditions injection of *C. parvum* increases the effectiveness of anti-tumour globulin in inhibiting the growth of the EL4 lymphoma in C57BL/6 mice. This *C. parvum* effect can be even more pronounced when the *C. parvum* is used in combination with anti-tumour drugs, and it should be noted that the drugs used represented groups with different mechanisms of action. *C. parvum* alone did not inhibit EL4 growth, and its ability to improve the effectiveness of anti-EL4 (with or without drug) was only clearly shown when both *C. parvum* and tumour cells were injected i.p. When *C. parvum* was injected i.p. it failed to influence the effectiveness of anti-EL4 Ig against s.c. injected tumour cells. I.v. injected *C. parvum* not only failed to increase the effectiveness of anti-EL4 Ig, but partially reversed it and, when given 10 days before i.p. injection of tumour cells, caused significant enhancement of tumour growth. This pattern of effectiveness, in which *C. parvum* has to be administered to the site of tumour growth, is reminiscent of its reported greater effectiveness against some tumours when given by intra-tumour injection (see Milas & Scott, 1978). It should, however, be noted that the treatment adopted in our experiments did not leave survivors with increased resistance to re-challenge with EL4 cells, as has been found by others after intra-lesional injection of *C. parvum* (*e.g.* Likhite & Halpern, 1974). It would be of interest to determine whether administration of *C. parvum* into the site of an s.c. EL4 tumour affects the anti-tumour activity of subsequent treatment with anti-EL4 Ig.

Previously reported anti-tumour effects of *C. parvum* have been attributed to activation of macrophages (Milas & Scott, 1978) and it seems likely that the ability of *C. parvum* to increase the effectiveness of anti-tumour globulin also involves macrophage activation (it has been reported (Fakhri & Hobbs, 1973) that transfer of normal mouse peritoneal macrophages can improve the effectiveness of tumour-directed antibodies). The detailed mechanism can only be a matter for speculation at present. The simplest explanation might be that locally activated macrophages are capable of killing antibody-sensitized tumour cells. It is, however, possible that stimulated production of some components of complement (Schorlemmer *et al.*, 1977) might permit a more effective complement-mediated lysis of the tumour cells (an attempt to improve the effectiveness of anti-EL4 Ig by transfer of fresh C57BL/6 serum as a source of complement was, however, unsuccessful—unpublished results). It should perhaps be emphasized that in these experiments *C. parvum* alone was ineffective, and that the effect which it augmented was due to the presence of anti-EL4 antibodies. *C. parvum* may therefore be acting by a mechanism which is different from that by which it normally inhibits tumour growth.

The role of the cytotoxic drugs in this system is also unclear. It is known that combined treatment with *C. parvum* and cytotoxic drugs can be beneficial (Houchens *et al.*, 1976) although the timing of the treatments may be critical (Currie & Bagshawe, 1970). Our results do not provide much encouragement for this approach, but do suggest that the apparent synergism between tumour-directed antibodies and a range of cytotoxic drugs (Davies & O'Neill, 1973; Davies, 1974) may be further improved by the use of an agent such as *C. parvum*. Clearly it is important to determine whether these effects can be demonstrated under conditions more closely related to clinical situations, and with tumour systems other than the EL4 model used in the present experiments.

We are grateful for the expert technical assistance of Mrs S. Buckham.

### REFERENCES

Adlam, C. & Scott, M. T. (1973) Lymphoreticular stimulatory properties of *Corynebacterium parvum* and related bacteria. *J. Med. Microbiol.*, **6**, 261.

Currie, G. A. & Bagshawe, K. D. (1970) Active immunotherapy with *Corynebacterium parvum* in murine fibrosarcomas. *Br. Med. J.*, i, 541.

DAVIES, D. A. L. & O'NEILL, G. J. (1973) *In vivo* and *in vitro* effects of tumour-specific antibodies with chlorambucil. *Br. J. Cancer*, **28** (Suppl. 1), 285.

DAVIES, D. A. L. (1974) The combined effect of drugs and tumour specific antibodies in protection against a mouse lymphoma. *Cancer Res.*, **34**, 1.

DAVIES, D. A. L., BUCKHAM, S. & MANSTONE, A. J. (1974) Protection of mice against syngeneic lymphomata: Collaboration between drugs and antibodies. *Br. J. Cancer*, **30**, 305.

FAKHRI, O. & HOBBS, J. R. (1973) Overcoming some limiting factors in tumour immunotherapy. *Br. J. Cancer*, **28**, 1.

HOUCHENS, D. P., JOHNSON, R. K., OVEJARA, A., GASTON, M. R. & GOLDIN, A. (1976) Effects of *Corynebacterium parvum* alone and in combination with adriamycin in experimental tumour systems. *Cancer Treat. Rep.*, **60**, 823.

KERBEL, R. A. (1979) Implications of immunological heterogeneity of tumours. *Nature*, **280**, 358.

LIKHITE, V. V. & HALPERN, B. N. (1974) Lasting rejection of mammary adenocarcinoma cell tumours in DBA/2 mice with intratumour injection of killed *Corynebacterium parvum*. *Cancer Res.*, **34**, 341.

LOWY, I., BONA, C. & CHEDID, L. (1977) Target cells for the activity of a synthetic adjuvant: muramyl dipeptide. *Cell. Immunol.*, **29**, 195.

MILAS, L. & SCOTT, M. T. (1978) Antitumour activity of *Corynebacterium parvum*. *Adv. Cancer Res.*, **26**, 257.

OETTGEN, H. F. (1977) Immunotherapy of cancer. *N. Engl. J. Med.*, **297**, 484.

PETO, R. & PIKE, M. C. (1973) Conservatism of the approximation $\Sigma(0-E)^2/E$ in the logrank test for survival data or tumour incidence data. *Biometrics*, **29**, 579.

REIF, A. E., LI, R. W. & ROBINSON, C. M. (1977). Passive immunotherapy of mouse leukemias with antisera of "directed" specificity: synergism with the action of cyclophosphamide. *Cancer Treat. Rep.*, **61**, 1499.

ROSENBERG, S. A. & TERRY, W. D. (1977) Passive immunotherapy of cancer in animals and man. *Adv. Cancer Res.*, **25**, 323.

RUBENS, R. D., VAUGHAN-SMITH, S. & DULBECCO, R. (1975) Augmentation of cytotoxic drug action and X-irradiation by antibodies. *Br. J. Cancer*, **32**, 352.

SALMON, S. E. (1977) Immunotherapy of cancer: Present status of trials in man. *Cancer Res.*, **37**, 1245.

SCHORLEMMER, H. U.. BITTER-SUERMANN, D. & ALLISON, A. C. (1977) Complement activation by the alternative pathway and macrophage enzyme secretion in the pathogenesis of chronic inflammation. *Immunology*, **32**, 959.

STEBBING, N. (1977) Effects of treatments with single-stranded polynucleotides on spontaneous AKR mouse leukaemia. *Leukaemia Res.*, **1**, 323.